# Membrane Type-1 Matrix Metalloproteinases and Tissue Inhibitor of Metalloproteinases-2 RNA Levels Mimic Each Other during *Xenopus laevis* Metamorphosis

**DOI:** 10.1371/journal.pone.0001000

**Published:** 2007-10-03

**Authors:** Logan A. Walsh, Deanna A. Carere, Colin A. Cooper, Sashko Damjanovski

**Affiliations:** Department of Biology, University of Western Ontario, London, Ontario, Canada; National Institute on Aging, United States of America

## Abstract

Matrix metalloproteinases (MMPs) and their endogenous inhibitors TIMPs (tissue inhibitors of MMPs), are two protein families that work together to remodel the extracellular matrix (ECM). TIMPs serve not only to inhibit MMP activity, but also aid in the activation of MMPs that are secreted as inactive zymogens. *Xenopus laevis* metamorphosis is an ideal model for studying MMP and TIMP expression levels because all tissues are remodeled under the control of one molecule, thyroid hormone. Here, using RT-PCR analysis, we examine the metamorphic RNA levels of two membrane-type MMPs (MT1-MMP, MT3-MMP), two TIMPs (TIMP-2, TIMP-3) and a potent gelatinase (Gel-A) that can be activated by the combinatory activity of a MT-MMP and a TIMP. In the metamorphic tail and intestine the RNA levels of TIMP-2 and MT1-MMP mirror each other, and closely resemble that of Gel-A as all three are elevated during periods of cell death and proliferation. Conversely, MT3-MMP and TIMP-3 do not have similar RNA level patterns nor do they mimic the RNA levels of the other genes examined. Intriguingly, TIMP-3, which has been shown to have anti-apoptotic activity, is found at low levels in tissues during periods of apoptosis.

## Introduction

**Figure 1:**
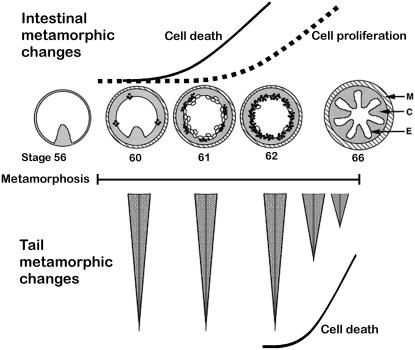
Schematic of Intestinal and Tail Cell Death and Cell Proliferation Events During Metamorphosis. *Xenopus laevis* metamorphosis begins when T3 levels elevate at stage 57 and terminates at stage 66 [26]. Top illustrations are of intestine cross-sections and are not to scale. Distances between stages are also not to scale. In response to T3 the intestine changes from a simple structure with one luminal fold (stage 56) to one with multiple folds in the post-metamorphic froglet following stage 66. As intestine metamorphosis begins ECM remodeling in the connective tissue (C) results in cell death (open circles) and cell proliferation (closed circles) of overlying epithelial cells (E). Bottom illustrations represent tail lengths during metamorphosis. Tail regression due to cell death begins at stage 62/63 and is complete by stage 66. Solid line = cell death, dashed line = cell proliferation. C = connective tissue, E = epithelial, M = muscle

Amphibian metamorphosis is a late developmental event that has been used to examine numerous processes including; cell signaling, receptor function, gene regulation, morphogenesis, and effects of environmental toxins [1]–[4]. While intricate, the entire metamorphic process is controlled by one molecule, thyroid hormone (T3). During *Xenopus laevis* metamorphosis all tissues are altered in some way, where structures are either created *de novo* (such as the limbs), removed completely (such as the tail), or remodeled (skin, the head and gills, and internal organs such as the intestine amongst others). This T3 dependent process is exemplified in the intestine (Figure 1) where embryonic epithelial cell death and adult epithelial cell proliferation facilitate the metamorphoses of an herbivorous tadpole into an omnivorous frog [5]. Removal of the tail, on the other hand, is achieved largely through apoptotic events late in the metamorphic process (Figure 1). While the ECM remodeling in both of these organs is facilitated by a similar array of molecules [6]–[9], the different cellular responses (proliferation vs. death) to this remodeling allow for the investigation of the possible functions of the molecules that remodel the ECM.

Amongst the important molecules that are modulated by T3 during metamorphosis are matrix metalloproteinases (MMPs) [10]. Indeed, the first MMP identified, collagenase, was isolated from the resorbing frog tail [11]. MMPs are a family of 25 proteins that function to cleave and remodel the extracellular matrix (ECM) and other cell surface proteins. Accordingly, MMPs influence most cellular functions during development and in a number of pathologies [12]–[15].

MMPs are tightly regulated at transcriptional and posttranslational levels. Most MMPs are secreted as inactive zymogens (pro-MMPs) that must to be activated extracellularly, often by other already active MMPs. Interestingly membrane-type MMPs (MT-MMPs), a family of six molecules, are activated intracellularly in the Golgi by furin [16]. Active MT-MMPs have been shown to play an important role at the cell surface in not only cleaving ECM molecules, but also in activating other pro-MMPs, a process which counter-intuitively involves tissue inhibitor of metalloproteinases (TIMPs).

TIMPs are a family of four secreted molecules that together can inhibit the activity of all MMPs [17], but also work with MT-MMPs in distinct stoichiometric ratios to activate other pro-MMPs near the cell surface. For example, two MT1-MMP molecules act with one TIMP-2 molecule to activate a pro-Gelatinase-A (Gel-A) molecule [18]. Gel-A (also known as MMP-2) is a powerful enzyme that cleaves a broad range of ECM and cell surface substrates and whose mis-regulation has been associated with a number of developmental anomalies and pathological conditions [19].

We have previously described the expression patterns of *X. laevis* MT3-MMP and TIMP-3 during early developmental stages. MT3-MMP is localized in anterior and neural structures, while TIMP-3 is more ubiquitously expressed. Ectopic expression of either gene by injection of mRNA into fertilized embryos resulted in death [15], [20], [21]. In this study we investigated the RNA levels of three MMPs (MT1-MMP, MT3-MMP, Gel-A) and two TIMPs (TIMP-2 and TIMP-3) during *Xenopus* metamorphosis. Though the embryonic expression of some of these molecules had previously been examined, frog metamorphosis allowed us the opportunity to see in particular how MT-MMPs and TIMPs might be regulated together in a developmental process that is controlled by one molecule–T3.

## Results and Discussion

### Natural metamorphosis of the intestine

**Figure 2:**
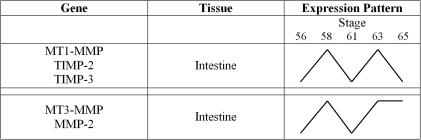
Patterns of MMP and TIMP RNA Levels Seen during *X. laevis* Intestine Metamorphosis Derived From RT-PCR Data. Levels of RNA found during intestine metamorphosis demonstrated that MT1-MMP, TIMP-2 and TIMP-3 shared similar RNA expression patterns, with peaks present at stages 58 and63. MT3-MMP and MMP-2 RNA expression patterns in the intestine did mirror each other, but were not similar to the three other genes. Changes in RNA levels are not quantitative, but instead illustrate patterns of expression.

**Figure 3:**
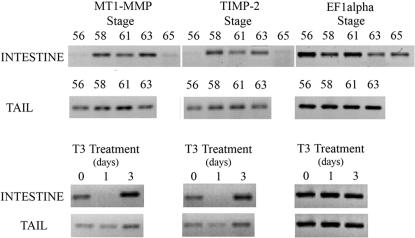
Representative RT-PCR Results of Similar MT1-MMP and TIMP-2 Levels of RNA During Natural and T3 Induced Metamorphosis. PCR products were agarose gel fractionated, stained, and photographed. MT1-MMP and TIMP-2 transcription patterns mimic each other at all stages and conditions. In the intestine, MT1-MMP and TIMP-2 transcripts are poorly detected at stage 56 prior to metamorphosis. Levels of both increase during stages 58, 61 and 63 and then drop to pre-metamorphic levels at stage 65. Conversely, in the tail, transcripts are detectable at stage 56, rise to stage 61, and then begin to drop at stage 63. T3 treatment induces a drop in levels after one day, but an increase after three days for both genes. EF1α RNA levels are shown for all comparable stages and treatments.

All five genes examined displayed increases in RNA levels as intestine metamorphosis commenced (Figure 2, stage 56 vs. 58). During intestine metamorphosis MT1-MMP, TIMP-2 and TIMP-3 RNA levels followed a general ‘M’ pattern, starting low, increasing, decreasing, increasing and then decreasing again as metamorphosis progressed (Figure 2). The first peak of RNA at about stage 58 corresponded to approximately when larval epithelial cells were beginning to undergo apoptosis (Figure 1). Several other MMPs (stomelysin-3, collagenase-3, collagenase-4 and MT1-MMP) have already been described to be expressed during intestine metamorphosis [3], [9], where MMP activation at this stage is involved in remodeling of the basement membrane resulting in the death of overlying cells [5]. Increased TIMP-2 RNA, in conjunction with MT1-MMP may also increase ECM remodeling by possibly activating Gel-A (whose RNA is also increased at this time). Indeed, when representative RT-PCR RNA levels of MT1-MMP and TIMP-2 are compared, they mirror each other in both the metamorphic intestine and tail (Figure 3).

The second peak of MMP and TIMP RNA levels in the intestine at about stage 63 correlates with the remodeling and cell proliferation associated with the morphogenesis of the multiple folds eventually seen in the post-metamorphic intestine (Figure 1). The continued high levels of RNA of MT3-MMP and Gel-A after this stage suggest that these two molecules, but not the other three genes examined here, play a role in later metamorphic remodeling events in the intestine.

### Natural metamorphosis of the tail

**Figure 4:**
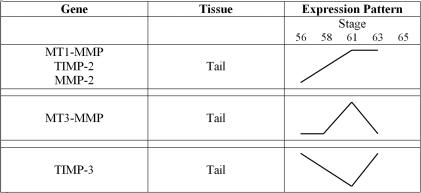
Patterns of MMP and TIMP RNA Levels Seen During *X. laevis* Tail Metamorphosis Derived From RT-PCR Data. Levels of RNA found during tail metamorphosis demonstrated that MT1-MMP, TIMP-2 and MMP-2 shared similar RNA expression patterns, peaking at stage 61 and staying elevated at stage 63. MT3-MMP and TIMP-3 expression patterns in the tail were not similar to the three other genes, nor to each other. Changes in RNA levels are not quantitative, but instead illustrate patterns of expression.

The RNA levels of the five genes were distinct during tail metamorphosis versus the metamorphic intestine (compare Figure 2 and Figure 4). MT3-MMP RNA displayed one peak at stage 61, while TIMP-3 RNA levels dipped at stage 61. MT1-MMP, TIMP-2 and Gel-A RNA levels increased as metamorphosis progressed and were maintained until the end of metamorphosis. Metamorphosis of the tail is a late event and one of the last to occur as all tail tissues are remodeled and removed in an efficient and safe manner. MMPs (Stromelysin-3, as well as collagenase-3 and –4) have already been shown to be expressed in distinct tail domains [3] and here high levels of MT1-MMP, MT3-MMP and Gel-A also support the role for these MMPs in tail resorption. The high levels of TIMP-2 supports two possible roles for this molecule. It can either work with MT1-MMP in activating other pro-MMPs, or conversely, if protein levels are high enough, TIMP-2 can inhibit and slow ECM remodeling to allow tail resorption to take place. TIMP-3 may then be playing an important role in modulating the speed of ECM degradation at late stages of metamorphosis (after stage 63), presumably a time when many MMPs are active and would otherwise degrade the ECM and induce apoptosis.

### Precocious T3 induction of metamorphosis

**Figure 5:**
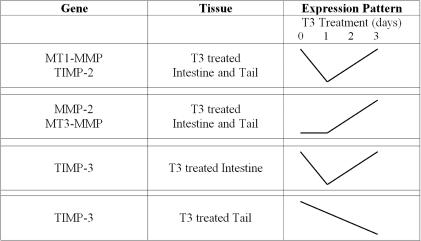
Patterns of MMP and TIMP RNA Levels Seen During T3 Induced Metamorphosis in *X. laevis* Intestine and Tail as Derived From RT-PCR Data. Levels of RNA found during induced metamorphosis of the intestine and tail demonstrated that MT1-MMP and TIMP-2 shared similar RNA expression patterns, dipping after 1 day and elevating thereafter. MMP-2 and MT3-MMP also shared similar RNA expression patterns, elevating after 1 day in both the intestine and tail. TIMP-3 expression patterns were not similar between the intestine and tail, nor were they similar to the four other genes. Changes in RNA levels are not quantitative, but instead illustrate patterns of expression.

In an effort to better understand the role of these five genes during metamorphosis, we examined if they were directly responsive to thyroid hormone induced metamorphosis. MMPs can contain thyroid hormone response elements in their DNA regulatory regions and can be transcribed directly by T3 and its receptor [22]. Genes that respond directly to T3 increase their transcription within hours. Our data suggest that while these genes are modulated during natural metamorphosis, we find that none of the genes we examined appear to be direct T3 responsive genes, as there was no increase in their RNA levels after 1 day of treatment (Figure 5). These studies also correlate well with other studies that observed an increase of transcription of four of these genes (MT1-MMP, MT3-MMP, TIMP-2 and Gel-A) by day three of T3 treatment [9], [23]. This suggests that there are numerous intervening players that are regulating their expression after the initial induction of metamorphosis by T3.

While four of our genes were eventually upregulated in the tail by the addition of T3, TIMP-3 RNA levels remained low (Figure 5). As the tail is one of the last tissues to undergo metamorphosis, it responds to T3 days after other tissues do during natural metamorphosis. It is likely that 3 days of T3 treatment is comparable to only the very early stages of tail metamorphosis. TIMP-3 levels do rise late in natural metamorphosis only after stage 61 (Figure 2 and Figure 4). However, this often takes many days after natural metamorphosis has started, and to expose tadpoles to high levels of exogenous T3 for long periods of time to induce complete tail regression is impractical, as it is most often lethal. Parenthetically, TIMP-3 levels may remain low during early stages as TIMP-3 has been shown to have anti-apoptotic properties [24], characteristics that would be detrimental to the regression of the tail.

### Conclusions

The RNA levels of MT1-MMP and TIMP-2 mirror each other during natural and induced metamorphosis of both the intestine and the tail. Their RNA patterns are closely matched by Gel-A, except during late intestine metamorphosis where Gel-A levels stay elevated. It is known that MT1-MMP and TIMP-2 can work together to activate Gel-A and our RNA data support this activation scenario. By comparison, MT3-MMP and TIMP-3 do not share similar RNA patterns during intestine metamorphosis, tail metamorphosis, nor in response to T3. This suggests that MMP and TIMP expression patterns during development should be considered collectively as certain pairings of these molecules appear to play significant roles together. While there appears to be a clear elevation of genes in response to apoptotic and cell proliferation events both in the intestine and tail, the level of active proteins and the exact cellular distribution of these proteins remains to be elucidated.

## Materials and Methods

### Animals and Treatments

Free-swimming *Xenopus laevis* tadpoles (Boreal Northwest) were raised in dechlorinated tap water. For precocious induction of metamorphosis, 10nM thyroid hormone (3,5,3′ triiodothyronine: T3) was added to the rearing water of stage 55 and 56 embryos. All tadpoles were staged according to Nieuwkoop and Faber [25]. Animal care met the principles and guidelines of the Canadian Council on Animal Care, and the University of Western Ontario Animal Use Sub-Committee.

### RNA Extraction and Semi-quantitative RT-PCR Analysis

Total RNA from *Xenopus* intestine and tail was isolated at stages 56, 58, 61, 63 and 65, using TRIzol (Invitrogen) as per the manufacturer's instructions. Stage 65 tail is very small and largely a-cellular and thus was not used. Total intestine and tail RNA was also isolated by the same means from T3-treated tadpoles prior to treatment (Day 0), 1 day, or 3 days after T3 treatment. RNA was treated with RNase-free DNase I (Fermentas) to remove any DNA contamination. RNA integrity was checked by visualizing the 18S and 28S ribosomal RNAs with gel electrophoresis. First-strand cDNA synthesis was performed on 5 µg of total RNA using SuperScript™ II Reverse Transcriptase (Invitrogen). PCRs were performed according to the manufacturer's instruction using Platinum® Taq DNA Polymerase (Invitrogen).

The *Xenopus* MMP and TIMP specific primers used were as follows: MT1-MMP, ^5′^ATGATGACCGCAGAGGAATC^3′^ and ^5′^TGGCAGATGTCAGGTCCATA^3′^; MT3-MMP, ^5′^CCATGGTCTGGCTCCCCTCA^3′^ and ^5′^GTGGACCATAAATTTTCT^3′^; Gel-A, ^5′^CGAGGAAACTGCAACACAGA^3′^ and ^5′^GATGGAGCAGGGGAAACATA^3′^; TIMP-2, ^5′^TCCCTGTTGCTATGCTTGTG^3′^ and ^5′^GACATCATTCCCATTGTCCA^3′^; TIMP-3, ^5′^GCAATAAGCTGCTGGGAATC^3′^ and ^5′^TCAGTTTCTCCCACCTCTCG^3′^. The EF1-α control primers; ^5′^CCTGAACCACCCAGGCCAGAT^3′^ and ^5′^GAGGGTAGTCAGAGAAGCTCTCCACG^3′^, were used as standards, and as they flanked an intron in genomic DNA, they also controlled for genomic DNA contamination. EF1-α PCR reactions were always run in parallel with all genes of interest.

Aliquots were taken at different cycles to ensure that the PCR products analyzed were from the log growth phase of the reaction. RT-PCR products were resolved on 1% agarose gels, stained with ethidium bromide, and visualized using the Bio-Rad Gel Doc 1000 system. The intensities of the unsaturated product bands were quantified using Bio-Rad Quantity One c. 4.4.0 software and compared to EF1-α. All experiments were repeated at least three times with consistent results, and the identity of the resulting amplicons was confirmed by sequencing at the Robarts Research Institute (London, ON). The ratio for each gene interest to EF1-α, at different stages and conditions, were compared and recorded as an increase or decrease compared to the previous developmental stage or treatment condition. The resultant graphs illustrated general trends of RNA level increase or decrease relative to EF1-α. The scale of the Y-axis is not standardized between genes and should not be used to quantify or compare RNA gene against each other.
